# Optimized Synthesis and Stabilization of Superparamagnetic Iron Oxide Nanoparticles for Enhanced Biomolecule Adsorption

**DOI:** 10.1021/acsomega.4c07371

**Published:** 2025-01-07

**Authors:** Wanderson Juvencio Keijok, Luis Alberto Contreras Alvarez, Angelo Marcio de Souza Gomes, Fabiana Vasconcelos Campos, Jairo Pinto de Oliveira, Marco Cesar Cunegundes Guimarães

**Affiliations:** † Federal University of Espírito Santo, Av Marechal Campos 1468, Vitória, ES 29.040 090, Brazil; ‡ Physics Institute, Federal University of Rio de Janeiro, Rio de Janeiro, Cidade Universitária, Rio de Janeiro 21941 972, Brazil

## Abstract

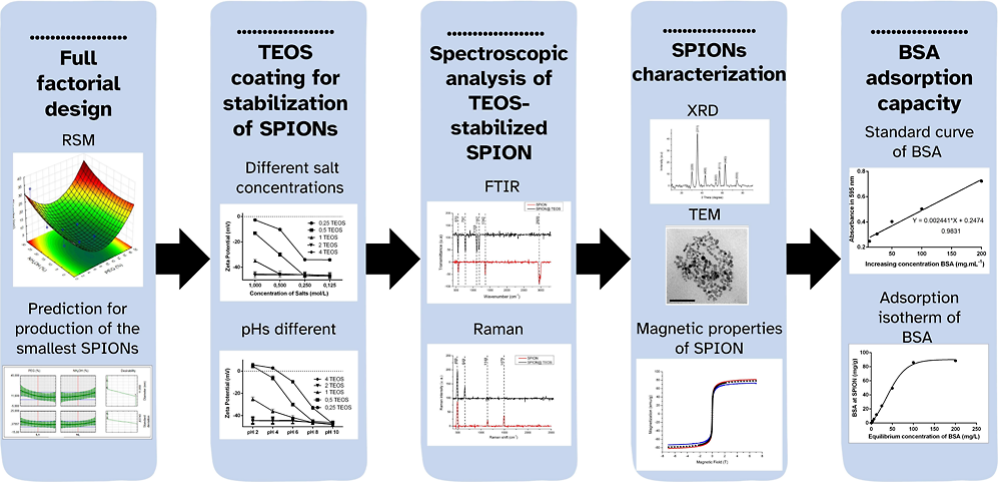

Monodisperse and colloidally stable magnetic iron oxide nanoparticles have been developed for diverse biotechnology applications. Although promising for the adsorption of organic molecules, the low density of adsorption sites in these nanoparticles has been a significant challenge. In this study, an optimized factorial design with response surface methodology (RSM) was employed to produce small Superparamagnetic Iron Oxide Nanoparticles (SPIONs) stabilized with tetraethoxysilane (TEOS). Bovine Serum Albumin (BSA) was selected for immobilization on the surface of SPIONs to test adsorption capacity. The model was validated by correlating significant factors with experimental responses, enabling the prediction of the smallest nanoparticle size. We obtained superparamagnetic SPIONs (75.12 emu/g) with high surface area and an average diameter of 11.06 ± 0.84 nm, with stability improved by the adsorption of TEOS (−46.24 mV) and suitable for pH values from 2 to 10 and salt concentrations up to 1 M. The maximum adsorption capacity of the nanoparticles was 87.8 ± 1.79 mg of BSA per gram of nanoparticles. The nanomaterial synthesized here presents a favorable platform for anchoring protein molecules via silanol groups on its electrostatically charged surface. This study introduces an effective strategy for the synthesis and stabilization of SPIONs with potential biotechnology applications.

## Introduction

Magnetic nanoparticles, renowned for their unique properties and multifunctionality, have been the focus of intense research and play a crucial role across a myriad of scientific and technological fields.^1^ In this context, superparamagnetic iron oxide nanoparticles (SPIONs) stand out, attracting significant attention due to their versatility and potential applications in fields ranging from medicine to biotechnology and beyond.^2−5^ The superparamagnetic behavior of SPIONs allows them to be readily magnetized in the presence of an external magnetic field and demagnetized when the field is removed.^6^ This intrinsic property allows for their efficient manipulation and separation through external magnetic fields, making them invaluable for purification and separation processes.^7^

The ability to functionalize nanoparticle surfaces is crucial for directing their interaction with specific biomolecules, enabling the development of highly selective and effective systems for biomedical and diagnostic applications.^8^ For instance, different groups of ligands have been put forward to increase the affinity of nanoparticles for proteins, improving their coating on the binding platform.^9−14^ The considerable surface area of SPIONs makes them highly suitable for functionalization and conjugation with biomolecules, offering a broad spectrum of potential applications, such as targeted drug delivery, diagnostic imaging, magnetic therapy, and biosensors.^15−17^

The synthesis method plays a crucial role in determining the final properties of SPIONs and thus significantly impacts their potential applications. Coprecipitation synthesis is a widely used technique recognized for its simplicity and efficiency in the large-scale production of magnetic nanoparticles.^18−20^ The ability to control nanoparticle size distribution and other parameters such as shape and functionalized surface by adjusting reaction conditions offers a significant advantage in producing SPIONs with specific properties for targeted applications.^18,19^ Therefore, the development of optimized experimental strategies, such as factorial design, is crucial. In fact, this topic has been widely discussed in the literature on SPIONs, especially regarding size control and physicochemical properties.^21^

The optimized Design of Experiments (DoE) strategy enables the systematic investigation of the effects of multiple factors on nanoparticle properties, resulting in a deeper, more accurate understanding of synthesis and functionalization processes. Through DoE, response surface methodology (RSM) can be performed to create an algorithm that enables the control of nanomaterials in an efficient, precise manner.^22^

Another crucial parameter is the size distribution of the magnetic nanoparticles produced using the coprecipitation method. This parameter can be controlled by adjusting reaction conditions, such as temperature, pH, time, and concentration of reactants, allowing the production of nanoparticles with specific sizes and size distributions for various applications.^23^

Currently, several types of SPION are commercially available, but their properties do not always meet the requirements for biomedical applications such as monodispersity, intrinsic magnetization, and surface functionality. In this study, we propose an approach based on factorial design to control the size of SPIONs and maximize the efficiency of protein adsorption on their surface. Bovine Serum Albumin (BSA) was selected for immobilization on the surface of SPIONs.

A thorough comprehension of the properties of SPIONs and their interaction with biomolecules is essential for advancing new applications and enhancing existing ones. Therefore, this study aims to advance the field of biomedical nanotechnology by providing valuable insights into the synthesis and functionalization of magnetic nanoparticles for biotechnology and medical applications, emphasizing the need for optimized strategies to develop safe and effective nanoparticles.

## Materials and Methods

### Materials

FeCl_2_·4H_2_O (Sigma-Aldrich 44939), FeCl_3_·6H_2_O (Sigma-Aldrich F2877), ammonium hydroxide 30% (Prochemios), polyethylene glycol-4000 (PEG-4000), tetraethyl orthosilicate (TEOS, 99%), argon gas (Oxivit, 99.999%). Ultrapure water (resistivity = 18.2 MΩ cm, Millipore Synergy Merck), neodymium magnet 50 × 50 × 12 mm (Supermagnet, Brazil). All glassware was sanitized with aqua regia (HCl/HNO_3_) and rinsed ten times with ultrapure water before the experiments.

### Experimental Design

To carry out the experimental design we used the STATISTICA 10.0 software. A Plackett-Burman design (16 experiments) was applied to identify the effect of five factors on one response (2^5–1^) that influences the desirable properties during SPION synthesis. In this screening, the factors were evaluated at two levels, maximum and minimum. The variables studied included molar ratio, time, temperature, NH_4_OH concentration, and polyethylene glycol concentration. The variable stirring speed was kept constant at 150 rpm/min. The levels of experimental factors used in this fractional factorial design are presented in Table 1.

**Table 1:** Levels of Experimental Factors Used in the Fractional Factorial Design^a^

factors	levels	
	–1	+1
molar ratio (Fe^3+^/ Fe^2+^)	1	3
time (min)	10	50
temperature (°C)	30	90
NH_4_OH concentration (%)	8	28
PEG 4000 concentration (%)	1	10

a (−1) low values, (+1) high values.

### Response Surface Method

Response surface method (RSM) is a set of mathematical techniques that describe the relationship between independent variables and one or more responses.^25,26^ The central composite design (CCD) was adopted using STATISTICA 10.0 to optimize the influence of the variables investigated (molar ratio, time, temperature, NH_4_OH concentration, and polyethylene glycol concentration).

The responses obtained from the CCD were subjected to second-order multiple regression analysis, as well as analysis of variance (ANOVA) with a 5% confidence level (*P* value < 0.05).

### Synthesis of SPIONs

Iron oxide nanoparticles were synthesized using the coprecipitation method, with varying molar ratios of FeCl_2_·4H_2_O and FeCl_3_·6H_2_O and using NH_4_OH as a precipitating agent. After the formation of a black precipitate consisting of Fe_3_O_4_ that exhibited magnetic properties at the end of the synthesis, the material was subjected to a magnetic field generated by a neodymium magnet for 2 min. The resulting supernatant was removed and the precipitate resuspended in ultrapure water. This washing procedure was repeated four times, aiming to ensure the complete removal of residues and impurities from the material obtained. The synthesis was conducted at different temperatures and times. In a subsequent step, PEG4000 was added at different times, maintaining the temperature of the formed colloid. The experimental conditions were determined through experimental design.

### Stabilizing SPIONs with TEOS Coating

After optimizing the smallest nanomaterial produced by the model, 1 g of the magnetic precipitate formed from Fe_3_O_4_ was resuspended in ethanol with 0.25%, 0.5%, 1%, 2%, and 4% TEOS to a final volume of 100 mL. The Fe_3_O_4_/TEOS ratio (g/mL) was 1:0.25, 1:0.5, 1:1, 1:2, and 1:4. The mixture was heated at room temperature for 24 h and the product thus obtained (Fe_3_O_4_@SiO_2_) was collected by a magnetic bar and washed ten times with ethanol and ultrapure water to remove impurities. Changes in colloid absorption were evaluated in different pH and ionic strength (NaCl) ranges, as in biological applications the stability of nanomaterials can be influenced by constant changes in these conditions in the environment.

### SPIONs Characterization

The average diameter of 500 nanoparticles was determined from images obtained by High Resolution Transmission Electron Microscopy (HRTEM) using a JEM-2100, Jeol, Tokyo, Japan, equipped with EDS, Thermo Scientific. The electron beam was generated by a lanthanum hexaborate (LaB_6_) filament, with acceleration voltages of 200 kV and 100 kV. The resolution for the 200 kV voltage was 2.5 Å. The images were captured with an ORIUSTM SC 1000 CCD camera, Gatan, and processed with DigitalMicrograph software. The colloids had their optical properties evaluated by UV–vis absorption spectrophotometry (Evolution§R 300 ThermoScientific). X-ray diffraction was conducted using the Phillips PW 1710 diffractometer (Cu k radiation).

Vibrating sample magnetometer (VSM) data measurements were performed using the MPMS SQUID 7.0. The stability (Zeta Potential) and Dynamic light scattering (DLS) of the nanomaterial were evaluated by the Microtac Zetatrac particle analyzer. Infrared spectroscopy and Raman Scattering measurements were performed in FTIR (FT-MIR FTLA 2000 Bomem) and Raman (ALPHA 300 R Confocal Raman Spectrometer) modes, respectively.

### BSA Adsorption on SPIONs

SPION-BSA bioconjugates were prepared by mixing a BSA solution with SPIONs. For the preparation of a series of bioconjugates with increasing BSA concentrations, the concentration of SPIONs was kept constant at 1 g, while that of BSA increased from 0 to 200 mg/L. The mixture was incubated at room temperature for 10 min under 150 rpm stirring.

After incubation, SPION-BSA conjugates were separated by magnetic decantation and washed three times with PBS to remove any unbound BSA. The BSA concentration in the SPION-BSA bioconjugates was measured by UV–vis spectrophotometry (Varian Cary 300 scan). An aliquot of the supernatant (10 μL) was then added to Bradford reagent (3 mL) and mixed for 2 min at room temperature, resulting in a blue color. BSA concentration was measured by absorption at 595 nm. The test was repeated three times for each sample and the average of the replicate measurements used to determine the equilibrium BSA concentration.

Maximum adsorption was determined and compared between different BSA concentrations, providing a detailed analysis of the conjugation process and adsorption efficiency in relation to the initial BSA concentration.

## Results and Discussion

### Full Factorial Design

The variables frequently used during the synthesis of magnetic nanoparticles: molar ratio, time, temperature, NH_4_OH concentration, and polyethylene glycol (PEG) concentration were analyzed using fractional factorial design (2^5–1^) (Table S1), in order to determine which were the most significant (*p* < 0.05) ones for the synthesis of SPIONs using diameter as the response variable (Table S1).^24−29^

The relationship between the variables studied and the size of the nanostructures are shown in Table 2. The analysis of variance (ANOVA) of the diameter reveals that the independent variables NH_4_OH concentration and PEG concentration had the greatest impact on the response, with a significance level of 0.05. Therefore, these variables were selected for the optimization of nanoparticle synthesis and construction of the full factorial design (3^2^). Further details on the choice of the most significant variables are available in the Supporting Information (Figure S1).

**Table 2:** Matrix of the Central Composite Face Design with the Most Significant Variables and Observed Response

assay	variables		response
	PEG (%)	NH_4_OH (%)	size (nm)
1	5.5 (0)	16.0	12.12
2	0.5 (−1)	16.0	17.78
3	5.5 (0)	28.0	13.71
4	10.5 (+1)	16.0	11.35
5	5.5 (0)	4.0	11.77
6	5.5 (0)	16.0	12.12
7	0.5 (−1)	4.0	13.78
8	0.5 (−1)	28.0	34.31
9	5.5 (0)	16.0	12.12
10	10.5 (+1)	28.0	11.60
11	10.5 (+1)	4.0	11.05

Previous studies corroborate our findings, emphasizing the critical influence of NH_4_OH and PEG concentrations on nanoparticle formation and its subsequent properties.^30−32^ NH_4_OH serves as both a precipitating agent and catalyst, supplying OH^–^ ions that react with Fe^2+^ and Fe^3+^ ions in solution to form iron hydroxides, which subsequently transform into iron oxide nanoparticles (Fe_3_O_4_) under suitable pH and temperature conditions. Additionally, NH_4_OH regulates the pH of the reaction, ensuring the formation of the magnetic Fe_3_O_4_ phase and stabilizing the synthesis environment.^33,34^ PEG binds to the surface of the Fe_3_O_4_ nanoparticles through hydrogen interactions between its hydroxyl groups and the oxygen atoms or iron ions on the nanoparticle surface. Additionally, van der Waals forces help maintain PEG adsorption, and when surface modifications are introduced, electrostatic interactions may also occur. This PEG layer not only regulates nanoparticle growth but also provides steric stabilization by forming a physical barrier that prevents aggregation, ensuring better colloidal dispersion.^28,35^ Therefore, maintaining the right balance between NH_4_OH and PEG concentrations is crucial for optimizing the size, shape, and stability of the nanoparticles, which are key factors for their successful application in biological environments.

### Optimizing the Production of Iron Oxide Nanoparticles

**Figure 1:**
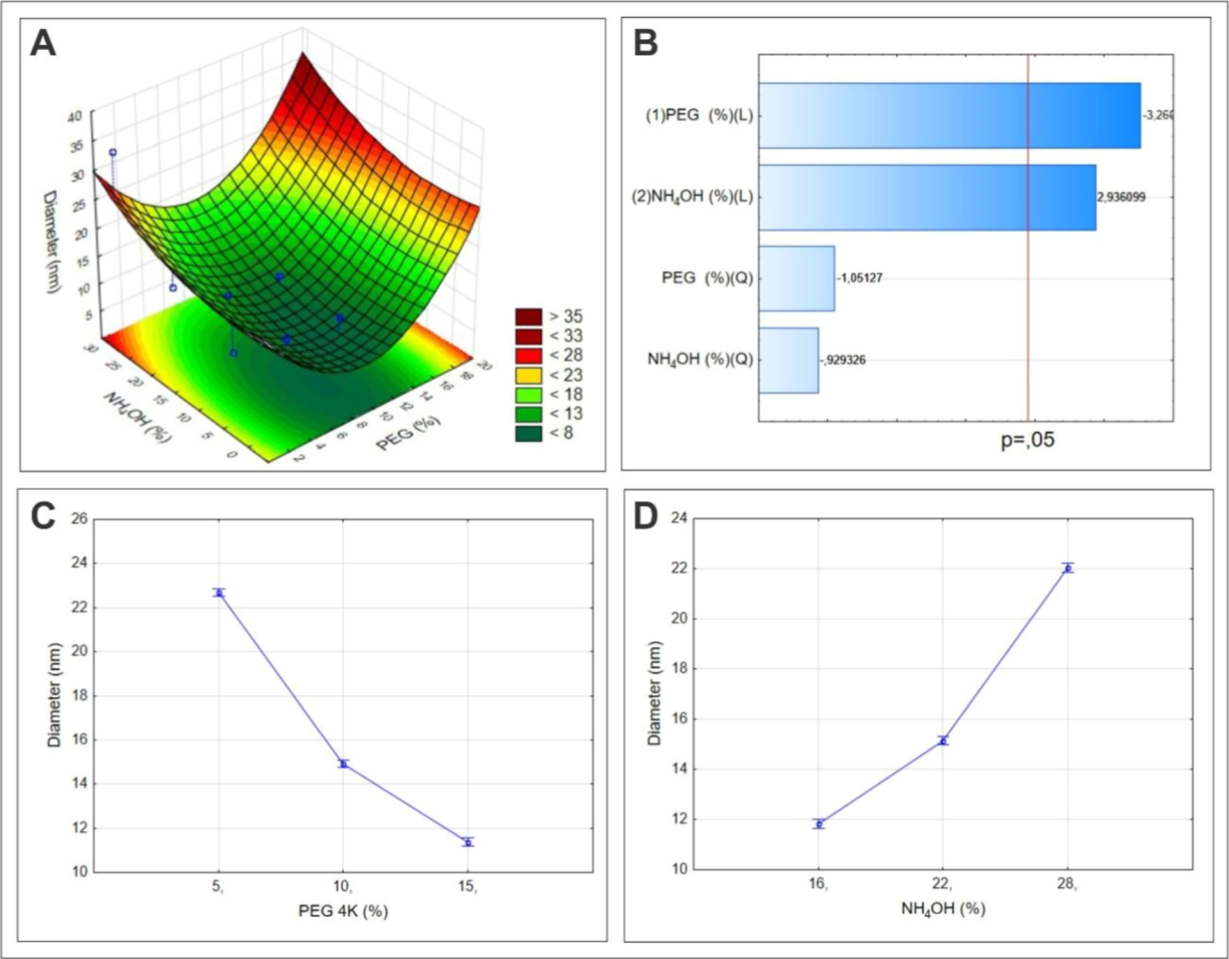
Design of experiments (DoE) analysis for magnetic nanoparticle size. (A) Response surface plot of the synthesized nanoparticles. (B) Pareto chart plotting the vertical lines that define significant variables (0.05 significance level). (D) Regression graph demonstrating the isolated effect of the variables PEG concentration (C) and NH_4_OH concentration (D) on the size of the synthesized nanomaterials.

The refinement of the response surface methodology (RSM) based on TEM measurements provides a deeper understanding of size control in nanomaterials.^36,37^ This model confirms that the nanoparticles respond noticeably to variations in PEG and NH_4_OH concentration (Figure 1A). Indeed, the Pareto chart shows that their curves intersect the reference line at 0.05, indicating their high statistical significance (Figure 1B). Subsequent regression analysis reinforces these observations, delineating the direct influence of PEG concentration on decreasing the size of magnetic nanomaterials (Figure 1C), while an increase in NH_4_OH concentration results in a corresponding increase in the size of these nanomaterials (Figure 1D).

It is worth noting that the manipulation of PEG and NH_4_OH plays an essential role in the applicability of nanomaterials produced for a desired purpose, as observed in previous studies^38,39^ Therefore, identifying the ideal conditions for optimizing the response, rather than simply characterizing the entire response surface, is critically important.

In addition to providing a visual representation of the results, the response surface approach allows the numerical quantification of the best response for the production of the desired nanomaterials, within the size limits established by the factorial design. This success is attributed to the algorithm developed during the statistical analysis, providing a solid basis for optimizing the nanomaterial production process at the desired scale.

Although the production of magnetic nanoparticles has been extensively investigated, few studies focus on the production of uniform and stable nanomaterials using the coprecipitation strategy. In this study, we sought to optimize the synthesis of magnetic nanoparticles by analyzing the reaction parameters to produce the smallest uniform and stable nanoparticles.^40−47^

### Predictive Analysis and Synthesis

**Figure 2:**
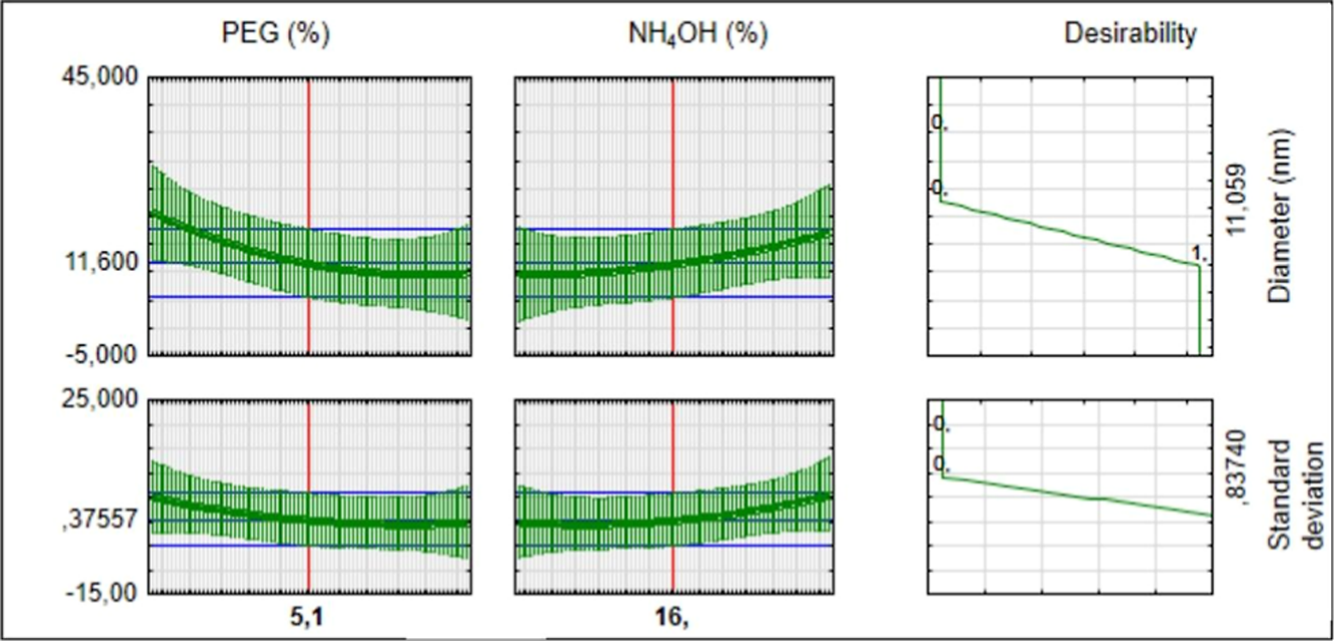
Prediction chart for production of the smallest iron oxide nanoparticles.

Predictive statistical analysis using factorial design data revealed that the combination of 16% NH_4_OH and 5.1% PEG results in the synthesis of nanoparticles with an average diameter of 11.06 ± 0.84 nm (Figure 2), the smallest nanoparticles obtained within the scope of our experimental design. This finding is particularly relevant, as smaller nanoparticles can present distinct physical and chemical properties, potentially expanding their applications in areas such as catalysis, medicine, and electronics.^2,3^

The accuracy of the prediction, evidenced by the low standard deviation of 0.83740 nm, highlights the robustness of the predictive model used. These results not only corroborate the validity of factorial design as a tool for the controlled synthesis of nanoparticles, but also establish a basis for future investigations aimed at exploring other combinations of reagents and experimental conditions. The ability to precisely adjust the size of nanoparticles is crucial for developing materials with tailored properties, reaffirming the importance of the methodology used in this study.

### Model Validation

**Figure 3:**
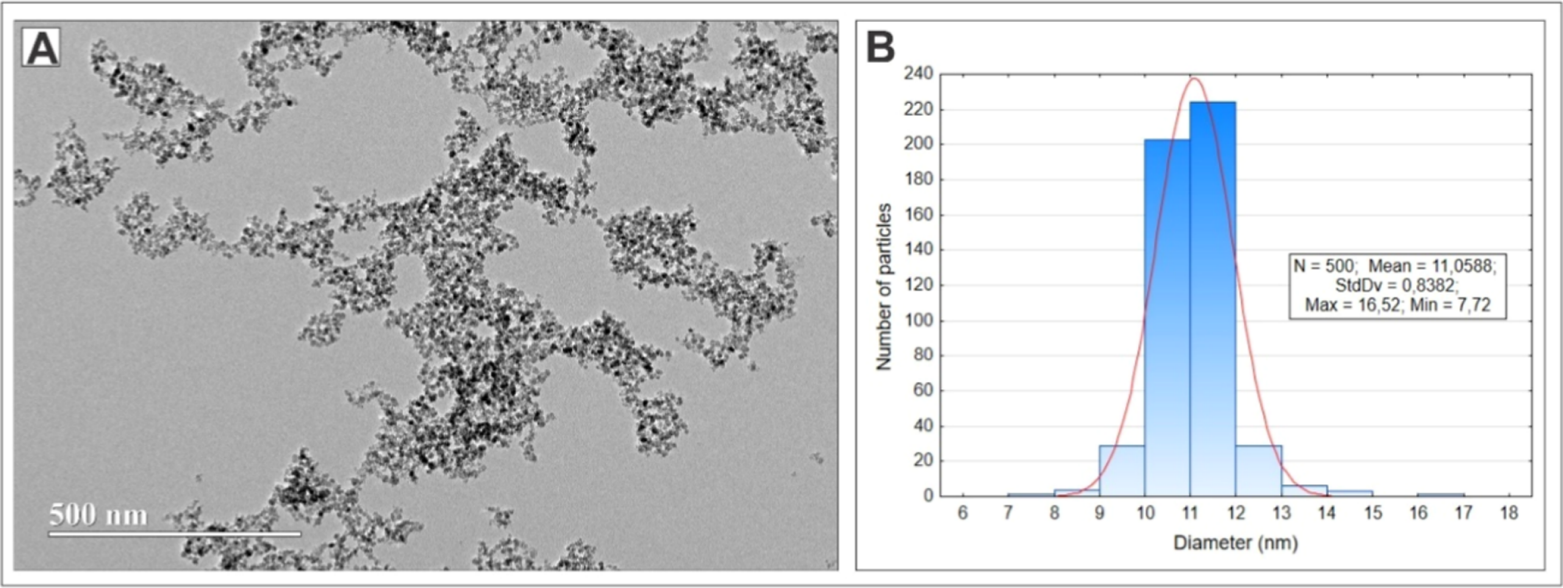
HRTEM images of SPIONs; Scale bar at 500 nm (A). Histogram showing the Gaussian distribution of the diameter of 500 particles analyzed by HRTEM (11.06 ± 0.84 nm) (B).

To validate the predictive parameters, the synthesis was performed under optimal conditions: 1.5 mL of 15 mM FeCl_3_ was mixed with 1.5 mL of 10 mM FeCl_2_ at room temperature for 5 min, followed by the addition of 1 mL of 5.1% PEG 4000 for another 10 min. After homogenization, 150 μL of 16% NH_4_OH was added to the mixture and incubated for 30 min at 60 °C. The colloid was cooled using a microwave bioreactor and subjected to magnetic separation for 5 min to remove unreacted substances. Using these parameters, monodisperse iron oxide nanoparticles were synthesized, presenting an average diameter of 11.06 ± 0.84 nm, as determined by counting 500 particles under high resolution transmission electron microscopy (HRTEM) (Figure 3). The narrow size distribution confirms the effectiveness of the predictive model. Statistical analysis demonstrated that the standard deviation of particle size was maintained within a narrow range, indicating the consistency and reproducibility of the synthesis process. These results highlight the ability to accurately predict and control nanoparticle characteristics, underlining the importance of factorial design in optimizing nanomaterial synthesis processes.

### Structural Characterization

**Figure 4:**
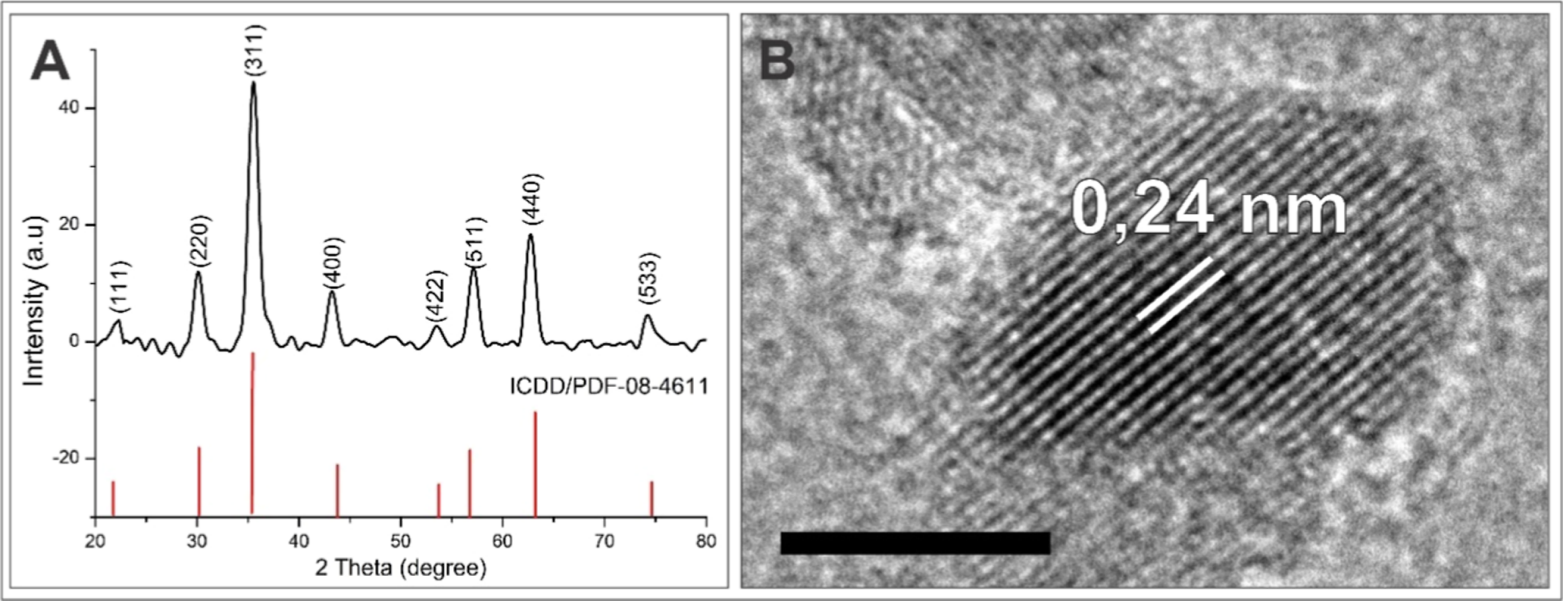
X-ray diffraction pattern of SPIONs (A); HRTEM images of SPIONs; Scale bar at 5 nm (B).

The formation of iron oxide nanocrystals was confirmed by X-ray diffraction (XRD), a crucial tool for the characterization of nanomaterials that allows the precise determination of the crystalline structure and the verification of the purity and quality of the synthesized nanoparticles. The X-ray diffraction patterns identified diffraction peaks with the following 2θ angulations: 30.20°, 36.50°, 38.20°, 42.91°, 53.80°, 57.30°, 63.21°, 71.90°, and 73.78°, which correspond to the crystal planes (220), (311), (222), (400), (422), (511), (440), (620), and (533) (Figure 4A). The HRTEM images confirmed the high crystallinity of the synthesized iron oxide nanoparticles, revealing a lattice spacing of 0.24 nm (Figure 4B), which aligns with the interplanar distances typical of spinel-like crystalline structures. The sharp definition of the observed peaks associated with this lattice spacing highlights the effectiveness of the synthesis method, demonstrating the successful production of iron oxide nanoparticles with the desired spinel crystalline structure.

### TEOS Coating for Stabilization of SPIONs

Assays carried out with nanoparticles in biological media often involve environments with varying pH and ionic strength, and it is essential to investigate the stability of these nanomaterials under these conditions.^48−50^ Preparing iron colloids with adequate stability is a complex process due to the inherent instability of these materials. In this study, we propose an optimized protocol using TEOS to coat the synthesized nanomaterials, aiming to preserve the initial structure of the nanomaterial and thus ensuring low aggregation.

Silica coating of nanoparticles using the Stöber method involves the hydrolysis and condensation of TEOS in the presence of a basic catalyst, typically NH_4_OH in an alcoholic medium. The hydrolysis of TEOS forms silanol groups (Si–OH), which then condense to create siloxane bonds (Si–O–Si), resulting in a uniform silica layer around the nanoparticle core. This layer enhances stability, and the Stöber process allows precise control over the silica thickness by adjusting the concentrations of TEOS, NH_4_OH, and the reaction time. This coating is crucial for protecting the iron oxide core from oxidation and improving colloidal stability, particularly in environments with varying pHs and ionic strengths, such as biological media.^51−53^

**Figure 5:**
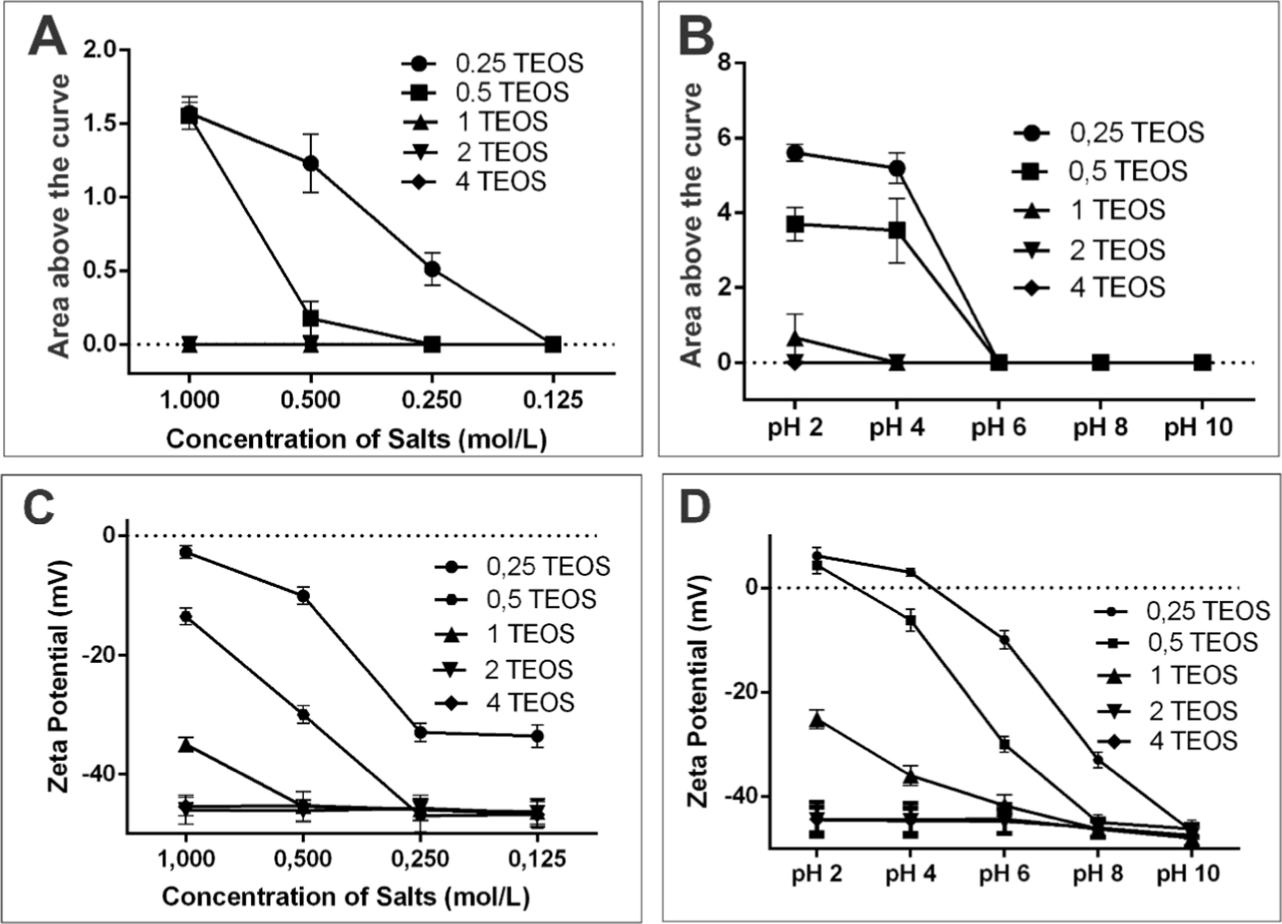
SPIONs functionalized with TEOS were evaluated under varying salt concentrations (A) and pH conditions (B). Zeta potential measurements of SPIONs functionalized with TEOS were performed under different salt concentrations (C) and pH conditions (D). All experiments were conducted in triplicate, and data are reported as mean ± standard deviation. Statistical significance was assessed, with all results demonstrating *p* < 0.05: (A) *p* = 0.0325, (B) *p* = 0.0404, (C) *p* = 0.0289, and (D) *p* = 0.0085.

Stability under different pH values and salt concentrations was analyzed in the supernatant after application of a magnetic field. To determine the best conditions for stabilizing the nanomaterials, we evaluated the following Fe_3_O_4_/TEOS ratios: 1:0.25, 1:0.5, 1:1, 1:2, and 1:4 (g/mL). The UV–vis spectrum in the 200 to 800 nm range revealed that the 1:2 ratio of Fe_3_O_4_/TEOS was the most effective for stabilizing the synthesized nanomaterials (Figure 5A,B). Furthermore, the zeta potential of the nanoparticles stabilized with the aforementioned ratio indicated an ideal surface charge to maintain the stable dispersion of the nanoparticles (Figure 5C,D), corroborating the UV–vis spectrum results. By confirming that TEOS-coated iron oxide nanoparticles are stable, we demonstrate that the coating not only preserves the initial structure of the nanoparticles but also provides a stable dispersion under varying pH and ionic strength conditions.

### Elementary Composition and Stabilization

**Figure 6:**
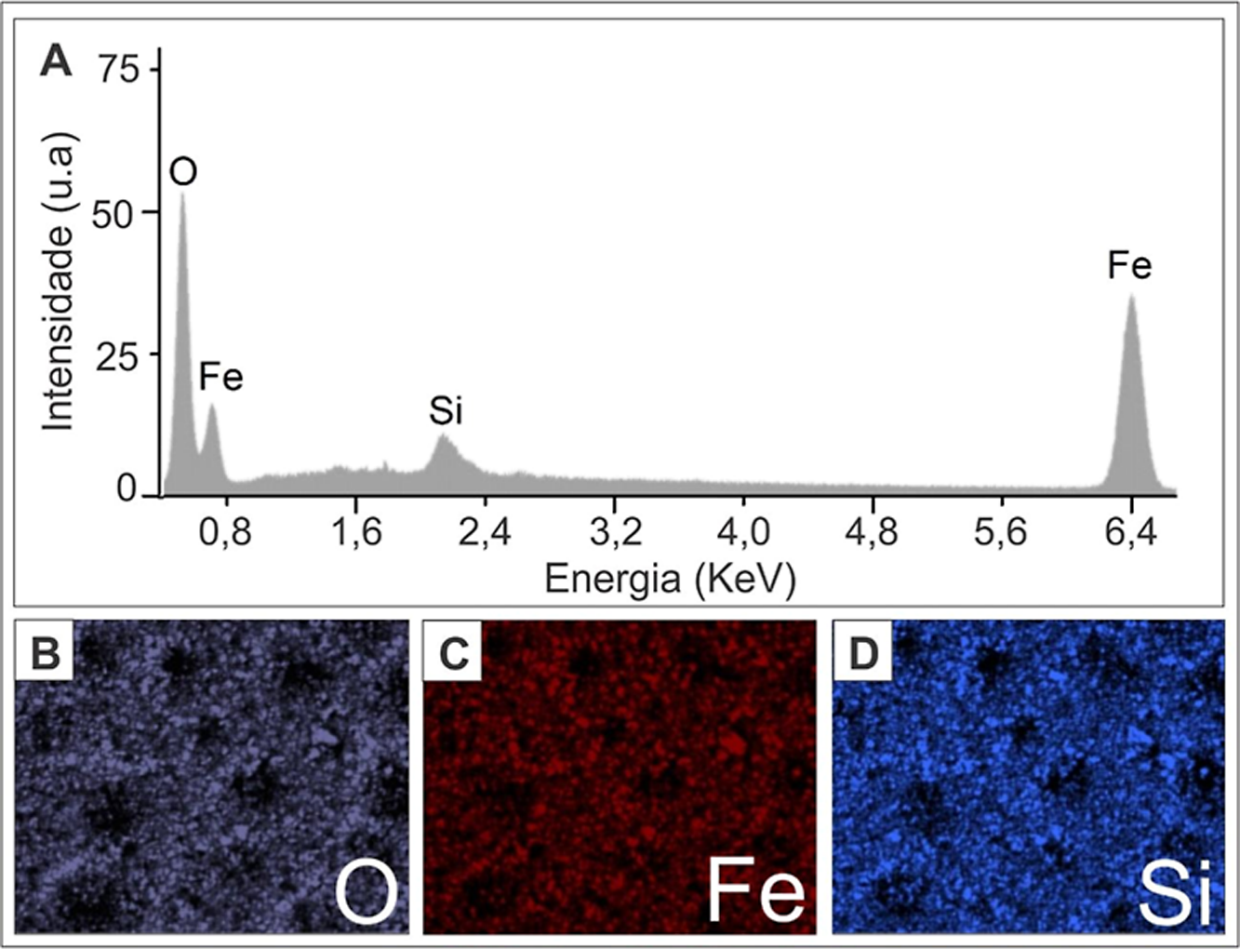
EDS analysis of TEOS-coated magnetic nanoparticles (A) and mapping images of elements corresponding to oxygen (B), iron (C) and silicon (D) in the sample.

Energy-dispersive X-ray spectroscopy (EDS) analysis was performed on the TEOS-stabilized iron oxide nanoparticles to determine their elemental composition (Figure 6A). The EDS spectrum revealed distinct peaks corresponding to oxygen (Figure 6B), iron (Figure 6C), and silicon (Figure 6D). The presence of a prominent Fe peak indicates the central iron oxide composition, while the O peak confirms its oxidized state. Furthermore, the Si peak reflects the effective coating of the nanoparticles with TEOS, providing the desired surface stabilization and functionality.

The elemental mapping analysis revealed a homogeneous distribution of Si on the surface of the nanoparticles, suggesting a uniform coating of TEOS. This uniformity is crucial to maintaining the stability and dispersibility of nanoparticles in various solvents.^7,54^ Additionally, Figure S2 shows the general EDS analysis, including all peaks, such as those for carbon and gold, which are related to the materials used during sample preparation. Therefore, the EDS results confirm not only the presence of the key elements in the nanoparticles, but also the successful implementation of the TEOS stabilization strategy.

### Magnetic Properties of Iron Nanoparticles

**Figure 7:**
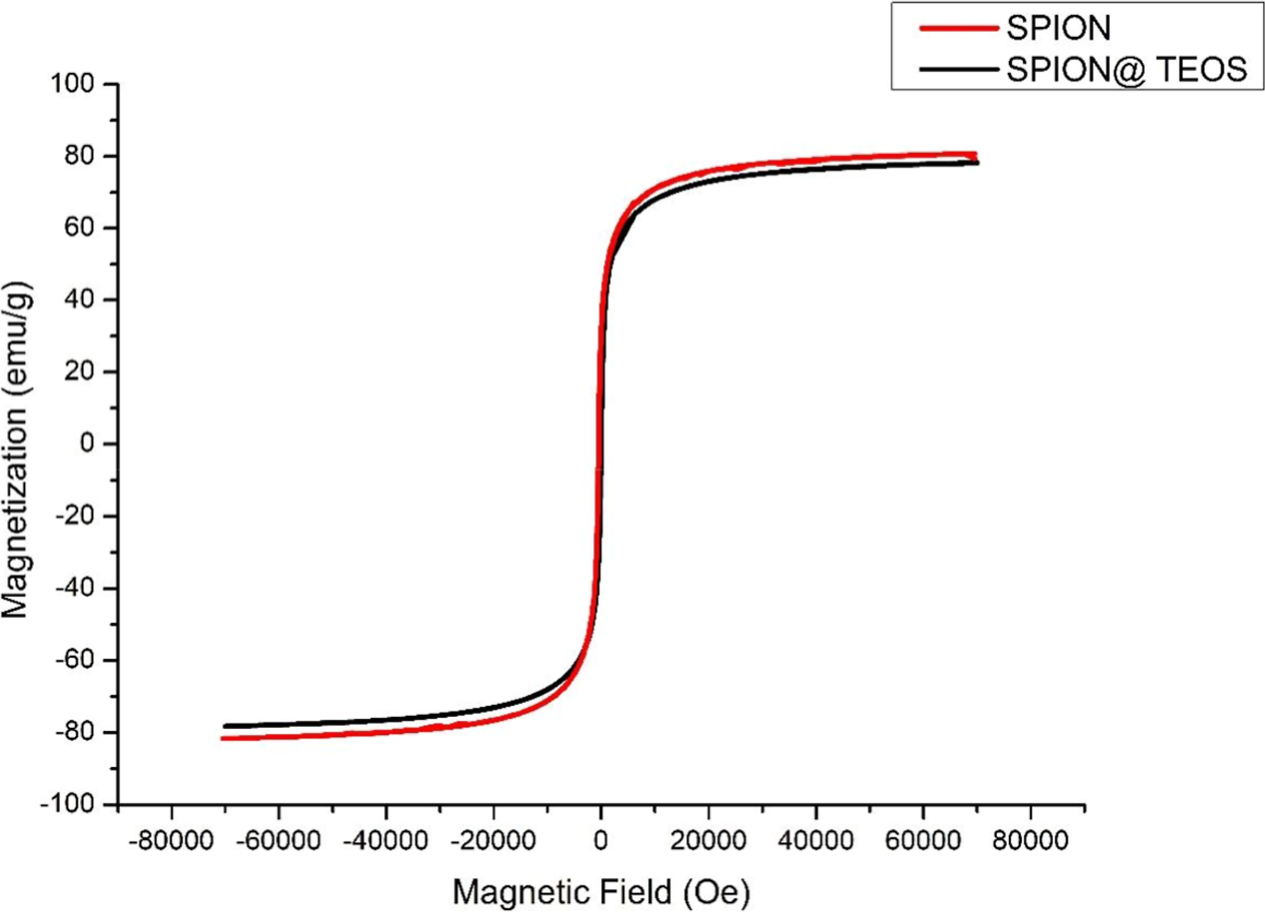
Magnetization of naked SPIONs and those with TEOS.

The magnetic properties of SPIONs are directly dependent on the size, size distribution, shape, and orientation of the particles.^55−58^ The magnetic properties of the SPIONs synthesized here were studied using a superconducting quantum interference device (SQUID). Figure 7 presents the magnetization curves (emu/g) for naked SPIONs and those with surfaces modified by the presence of TEOS. The magnetization remained practically unchanged at 75.12 emu/g, suggesting that surface coating with TEOS does not significantly affect the intrinsic magnetic properties of SPIONs (Figure 7). The stability of magnetic properties, even upon surface modification, is a desirable characteristic for applications in which surface functionalization is required without compromising the magnetic efficiency of nanoparticles. These findings corroborate the literature, which suggests that the magnetization of SPIONs is influenced by factors such as the nanoparticle nucleus and can sometimes be altered by surface modifications.^59−61^ However, our study demonstrates that coating with TEOS did not cause significant changes in the magnetization of SPIONs. In short, TEOS-coated SPIONs maintain their magnetic properties, regardless of the increase in hydrodynamic size, demonstrating the effectiveness of the functionalization methods used and the robustness of the magnetic properties of iron oxide nanoparticles.

### Spectroscopic Analysis of TEOS-Stabilized Iron Oxide Nanoparticles

**Figure 8:**
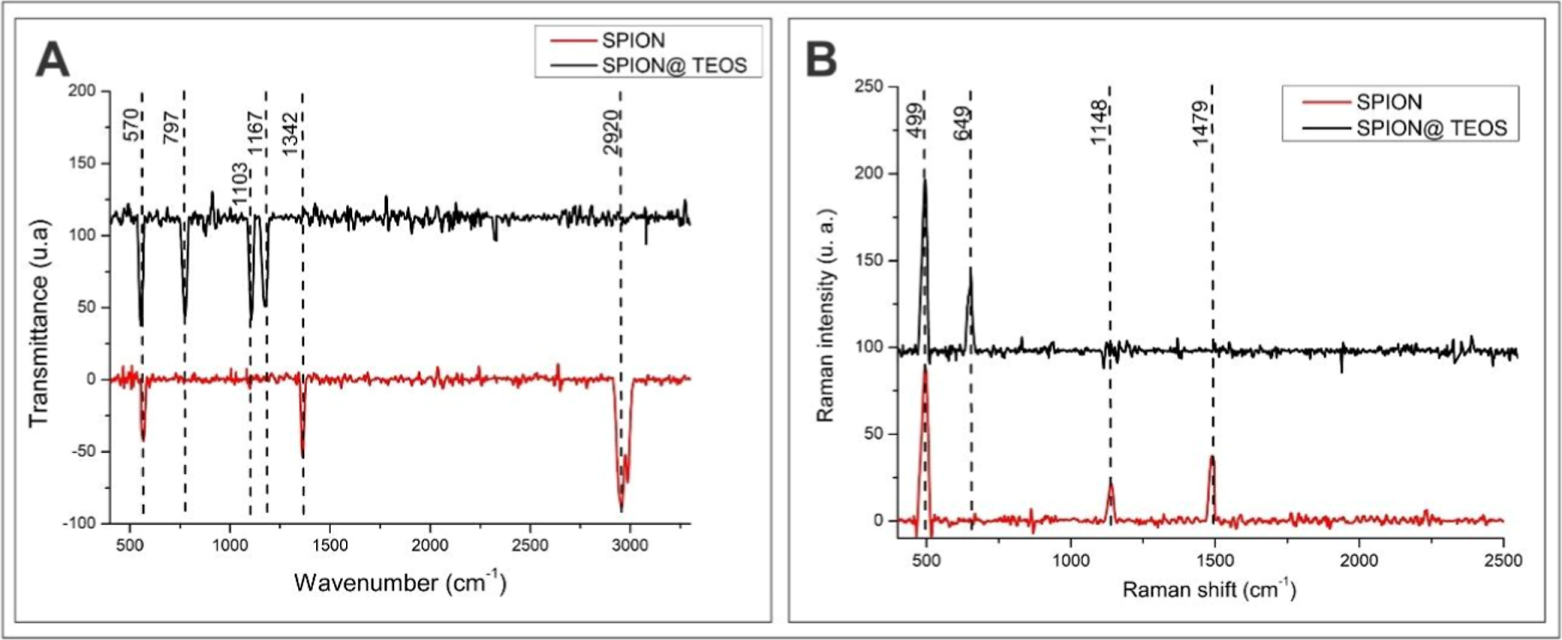
Comparison of FTIR (A) and Raman (B) spectra of SPIONs and SPIONs@TEOS.

The structural features of TEOS-stabilized iron oxide nanoparticles were analyzed by FTIR and Raman spectroscopy, which identified distinct absorption bands attributed to different functional groups present in the materials (Figure 8).

The FTIR bands (Figure 8A) at 570 and 390 cm^–1^ can be attributed to the Fe–O stretching mode of the tetrahedral and octahedral sites and the Fe–O stretching mode of the octahedral sites, respectively.^62^ Those around 2888, 2920, and 2888 cm^–1^ can be attributed to the alkyl chain of PEG, while the bands at 1342 and 1100 cm^–1^ are due to C–H bending and C–O stretching vibrations.^63^ TEOS peaks include 1167 cm^–1^ for Si–O–Si, 1103 cm^–1^ for Si–O–C, and 797 cm^–1^ for Si–O vibrations.^64^

Characteristic Raman peaks were also identified (Figure 8B), contributing to a comprehensive characterization of the nanoparticles. For instance, the band observed at 499 cm^–1^ is characteristic of maghemite (g-Fe_2_O_3_).^65^ The C–C stretching at 1148 cm^–1^, the CH_2_ rocking vibration, and the CH_2_–CH_2_ symmetric bending vibration at 1479 cm^–1^ are attributed to PEG.^66^ In addition, the presence of TEOS was confirmed by the peak at 649 cm^–1^ (SiO_4_^–^).^67^ These results further reinforce the composition and structure of the iron oxide nanoparticles functionalized with TEOS, validating the effectiveness of the synthesis protocol.

**Figure 9:**
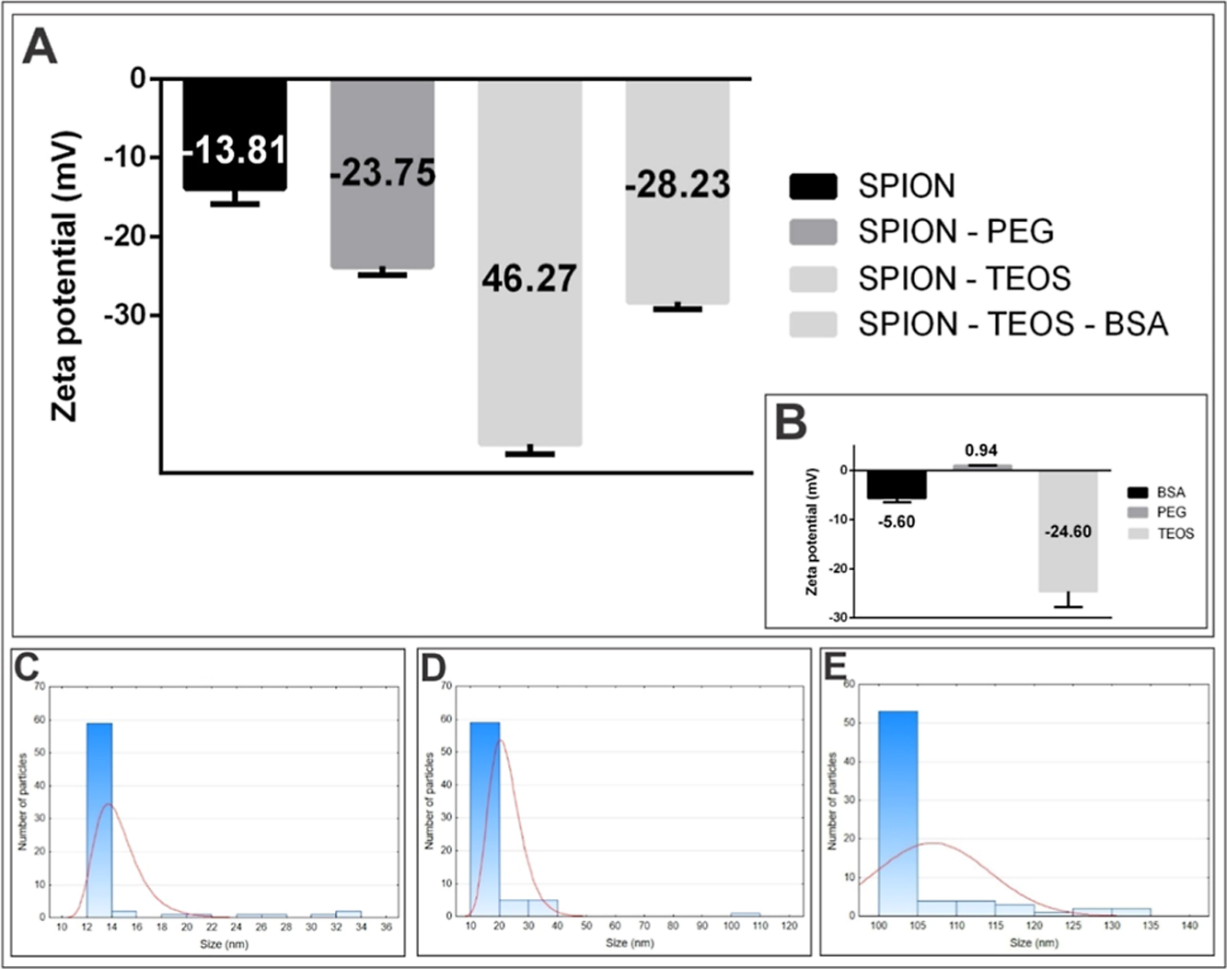
Zeta potential of SPIONs, SPIONs@PEG, SPIONs@TEOS, and SPIONs@TEOS@BSA, measured as −13.81 ± 2.02, −23.75 ± 1.06, −46.27 ± 1.31, and −28.23 ± 0.90 mV, respectively (A). Zeta potential of PEG (−5.61 ± 0.88 mV), BSA (0.95 ± 0.11 mV), and TEOS (−24.60 ± 3.17 mV) (B). DLS plot showing the size distribution of nanoparticles, with SPIONs@PEG (C), SPIONs@TEOS (D), and SPIONs@TEOS@BSA (E).

The comparison of the zeta potentials of SPIONs and SPIONs@TEOS revealed a significant change in colloidal stability and surface charge. Nonfunctionalized iron oxide nanoparticles exhibited a zeta potential of −13.81 ± 2.02 mV, while the zeta potentials of functionalized nanoparticles were measured as follows: SPIONs with PEG showed −23.75 ± 1.06 mV, SPIONs with TEOS showed −46.27 ± 1.31 mV, and SPIONs with BSA had −28.23 ± 0.90 mV (Figure 9A). For the reagents used, the zeta potential was −5.60 ± 0.88 mV for BSA, 0.94 ± 0.11 mV for PEG, and −24.60 ± 3.17 mV for TEOS (Figure 9B). It is worthy of note that after functionalization with TEOS, the zeta potential of the coated nanoparticles suffered a considerable increase, suggesting a reduction in electrostatic repulsion and possible aggregation due to lower surface charge. This change in zeta potential corroborates the formation of a TEOS layer on the surface of the nanoparticles, directly influencing their colloidal properties. This coating increases the hydrodynamic diameter compared to the diameter observed by TEM, which revealed an average diameter of approximately 12 nm for the iron oxide nanoparticles. In contrast, DLS measurements for the different SPION modifications showed a hydrodynamic diameter of 13.56 ± 0.74 nm for SPIONs@PEG, 21.32 ± 3.2 nm for SPIONs@TEOS, and 103.3 ± 10.4 nm for SPIONs@TEOS@BSA. The difference between the diameters obtained by TEM and DLS is attributed to the adsorption phenomenon, where the presence of PEG, TEOS, and BSA molecules on the nanoparticle surface increases the hydrodynamic radius, as observed in Figure 9C,D,E.

Although these optimized SPIONs demonstrate significant biomedical potential, it is essential to consider the toxicity and environmental impact associated with their use. Studies indicate that the toxicity of iron oxide nanoparticles may vary depending on size, prolonged exposure, concentration, and surface functionalization. In this work, we used TEOS as a stabilizing agent. This molecule is inert and consequently significantly reduces the toxicity of these SPIONs. However, we believe that future studies should focus on assessing biocompatibility and biodegradability to develop safe and sustainable approaches that maximize their therapeutic and diagnostic benefits while minimizing environmental risks.^68−70^

### BSA Adsorption Capacity

**Figure 10:**
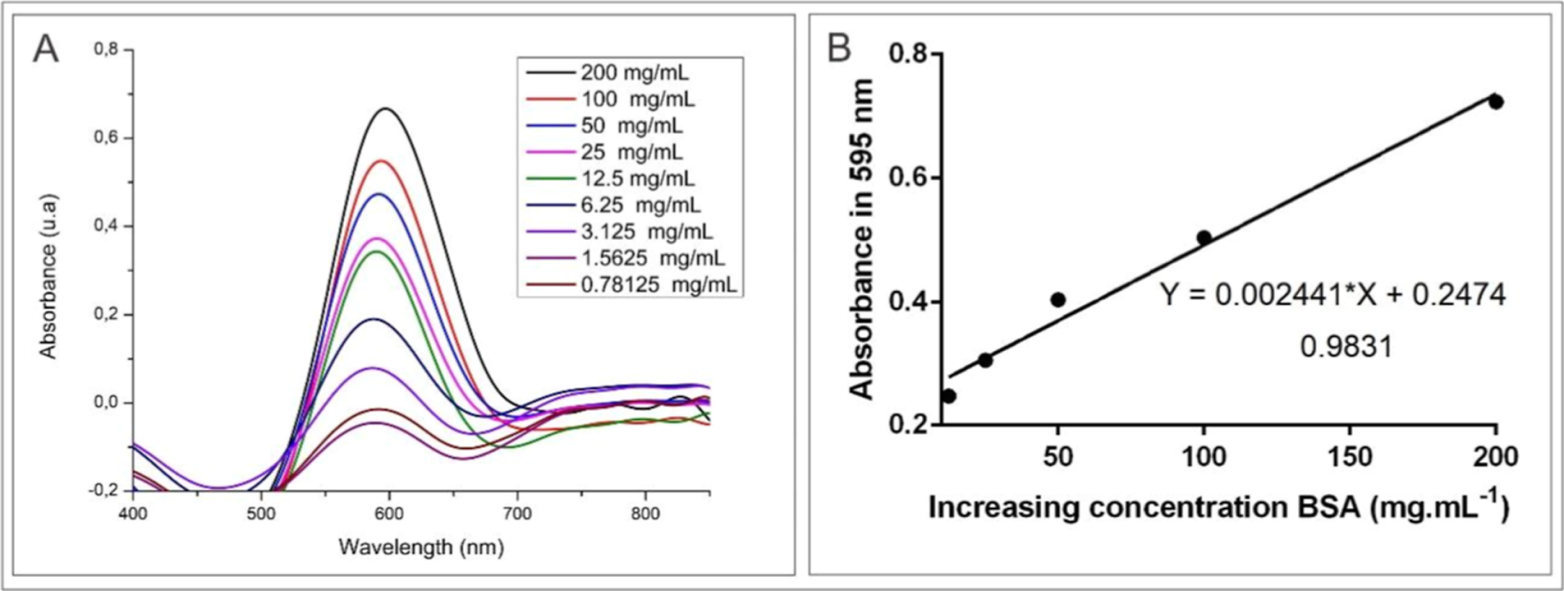
BSA standard calibration curve. Optical density of BSA (A). Standard curve of BSA concentration (B).

The adsorption capacity of the synthesized iron oxide nanoparticles was investigated using BSA as a model protein.^71,72^ Different concentrations of BSA were tested and the resulting standard curve showed a coefficient of determination (*R*^2^) of 0.9831, indicating high precision in the experimental data (Figure 10).

**Figure 11:**
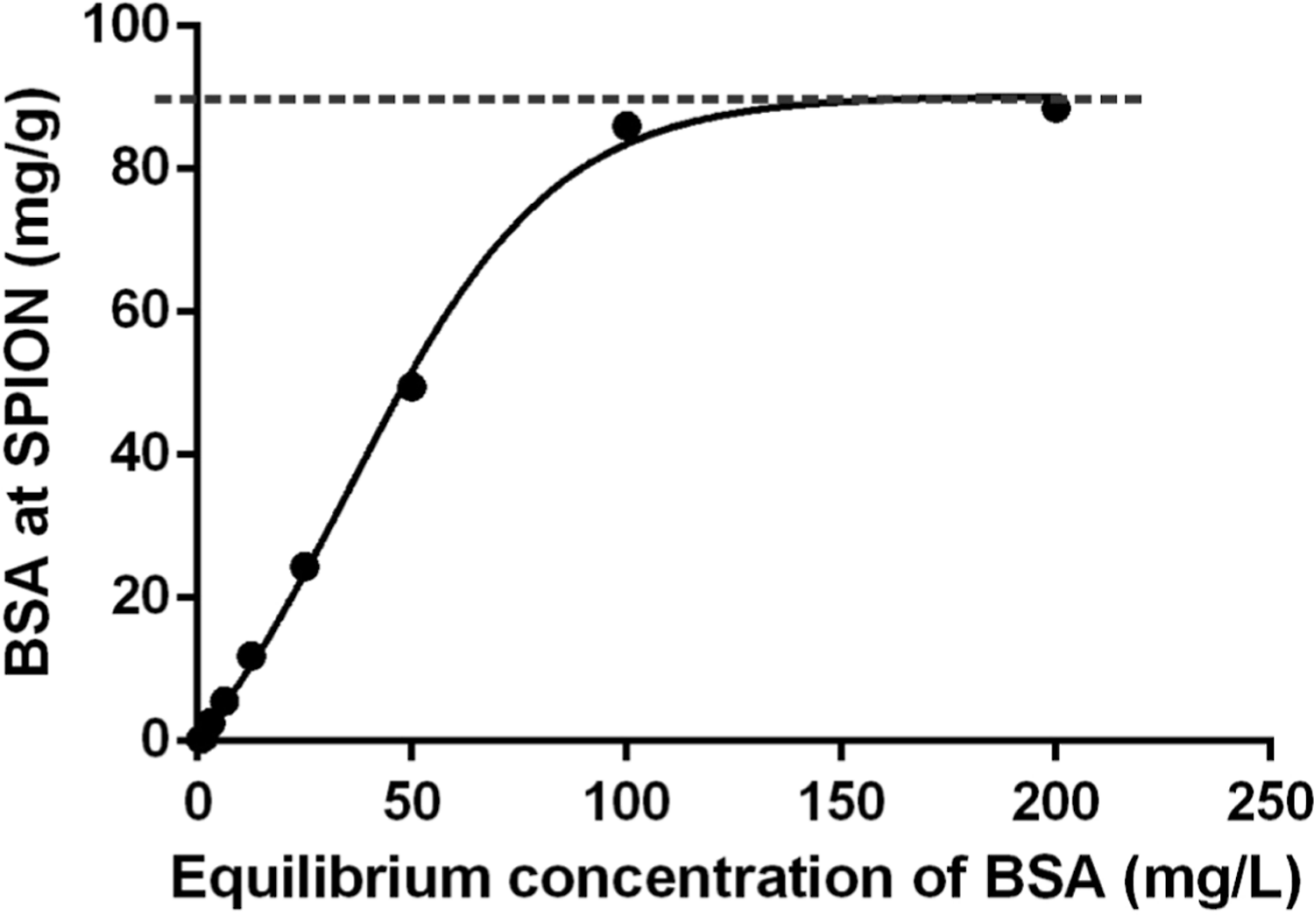
Langmuir adsorption isotherm of BSA on the surface of 1 g of SPIONs. The curve represents the adsorption capacity of BSA as a function of the equilibrium concentration, adjusted to the Langmuir model.

To better understand the binding mode of the protein to the functionalized nanostructures, BSA adsorption was performed under adjusted pH conditions to promote electrostatic interactions.^73,74^ The nanostructures were transferred to PBS and the pH was adjusted to 4.0, favoring the electrostatic bond between BSA and the iron oxide nanoparticles. The maximum adsorption capacity in this study was 87.8 ± 1.79 mg of BSA per gram of SPIONs, surpassing the previously reported value of 85 mg/g.^75^ This improvement can be attributed to factors such as process optimization and surface modification of the nanoparticles, which enhance their interactions with BSA. The analysis was performed using UV–vis spectroscopy, and the adsorption data were fitted to the Langmuir model.^76^ The Langmuir diagram (Figure 11) depicts an isotherm that indicates a monolayer adsorption.

These results demonstrate that iron oxide nanoparticles have a significant ability to adsorb proteins, a crucial feature for biomedical applications such as drug delivery systems and contrast agents in magnetic resonance imaging.^77,78^ The high adsorption capacity of BSA suggests that these nanoparticles may be effective in biological environments, where protein adsorption may influence the biodistribution and biocompatibility of nanoparticles.^79−81^ These findings highlight the potential of iron oxide nanoparticles as versatile platforms for the adsorption and delivery of biomolecules in therapeutic and diagnostic applications.^82,83^

## Conclusions

This study contributes to the advancement of the controlled and reproducible synthesis of iron oxide nanoparticles designed for biological applications. Through a fractional factorial design (2^5–1^) and a full factorial design (3^2^), the main synthesis parameters in the coprecipitation process were identified and optimized, resulting in monodisperse nanoparticles with an average diameter of 11.06 ± 0.84 nm. The nanoparticles exhibited a spinel crystalline structure, superparamagnetic properties (75.12 emu/g), and stability (−31.24 ± 1.14 mV) in solution. Stability was further enhanced by TEOS adsorption, achieving a zeta potential of −46.27 ± 1.31 mV, and the nanoparticles maintained their structural and colloidal stability across a pH range of 2 to 10 and ionic strengths from 0.125 to 1 mol/L NaCl, as demonstrated by UV–vis and zeta potential analyses.

Moreover, we demonstrated a high BSA adsorption capacity (87.8 ± 1.79 mg/g) compared to other studies, highlighting the potential of optimized SPIONs for drug delivery and diagnostic applications. Due to their high stability and magnetic properties, these iron oxide nanoparticles are well-suited for multifunctional theranostic uses, with implications for targeted drug delivery, real-time imaging, and diagnostic precision in complex biological environments. Thus, we believe this work underscores the importance of synthesis optimization as a key criterion for the application of SPIONs in various biotechnological fields.

## Supporting Information Available

Additional experimental and statistical analyses are provided; The 2^5–1^ fractional factorial design was used to identify the significant variables NH_4_OH and PEG in the nanoparticle synthesis, with the 16 assays described in Table S1; The effects analysis (Figure S1) and ANOVA (Table S2) highlight the significant factors for controlling the average diameter; Furthermore, optimization was performed using the central composite design (CCD), with NH_4_OH and PEG levels defined in Table S3, and the results validated by model fitting in Table S4; The EDS analysis (Figure S2) confirmed the main elements—iron, oxygen, and silicon—along with secondary peaks related to sample preparation, supporting the effectiveness of the stabilization strategy with TEOS (PDF)
