# Supplementary material for: Optimized Synthesis and Stabilization of Superparamagnetic Iron Oxide Nanoparticles for Enhanced Biomolecule Adsorption
Source: ACS Omega. 2025 Jan 7;10(2):1976–87. doi: 10.1021/acsomega.4c07371 (PMC11755185; doi:10.1021/acsomega.4c07371)
Supplement: Supplementary file 1 — ao4c07371_si_001.pdf [file ao4c07371_si_001.pdf]

Optimized Synthesis and Stabilization of Superparamagnetic Iron Oxide Nanoparticles for Enhanced Biomolecule Adsorption

Wanderson Juvencio Keijok[1], Luis Alberto Contreras Alvarez[1], Angelo Marcio de Souza Gomes[2], Fabiana Vasconcelos Campos[1], Jairo Pinto de Oliveira[1] and Marco Cesar Cunegundes Guimarães[1*]

[1] Federal University of Espírito Santo, Av Marechal Campos 1468, Vitória, ES 29.040-090, Brazil

[2] Physics Institute, Federal University of Rio de Janeiro, Rio de Janeiro, Cidade Universitária, Rio de Janeiro 21941-972, Brazil

*Corresponding authors:

Marco Cesar Cunegundes Guimarães - Federal University of Espírito Santo, Av Marechal Campos 1468, Vitória, ES 29.040-090, Brazil; Email: marco.guimaraes@ufes.br

Supplementary Material

Experimental design

Based on a review of the literature, several factors that interfere with the synthesis of superparamagnetic nanoparticles with a large surface area were identified. Given the numerous factors and levels that influence changes during synthesis, the primary factors and levels were selected based on the literature review (Table S1). The 2^{5-1} fractional factorial design was chosen to select the most significant variables, investigating the diameter of the nanomaterials produced (Table S2). After analyzing the experimental factors and levels, the two most significant variables for optimization were identified. Consequently, three levels and two factors were selected for the full factorial experimental design (3^2).

Table S1. Fractional factorial design matrix with the variables studied for the 16 syntheses of magnetic iron oxide nanoparticles.

Assay number	Studied variables					Response
	Temperature	Time	NH_4OH concentration	Molar ratio Fe^{2+}/Fe^{3+})	PEG concentration	Average diameter (nm)
1	30	10	8	1	10	7.60
2	90	10	8	1	1	9.94
3	30	50	8	1	1	10.17
4	90	50	8	1	10	8.96
5	30	10	28	1	1	23.48
6	90	10	28	1	10	11.34
7	30	50	28	1	10	13.95
8	90	50	28	1	1	39.28
9	30	10	8	3	1	10.60
10	90	10	8	3	10	9.75
11	30	50	8	3	10	8.63
12	90	50	8	3	1	11.63
13	30	10	28	3	10	10.67

14	90	10	28	3	1	28.05
15	30	50	28	3	1	39.28
16	90	50	28	3	10	13.26

The synthesis of nanoparticles was optimized based on the most significant effects of the fractional factorial design in five parameters, in order to determine the most relevant variables ($p = 0.05$), using size as a response. Additionally, the significance of the factors can be investigated by observing the distance of the significant factors in relation to zero

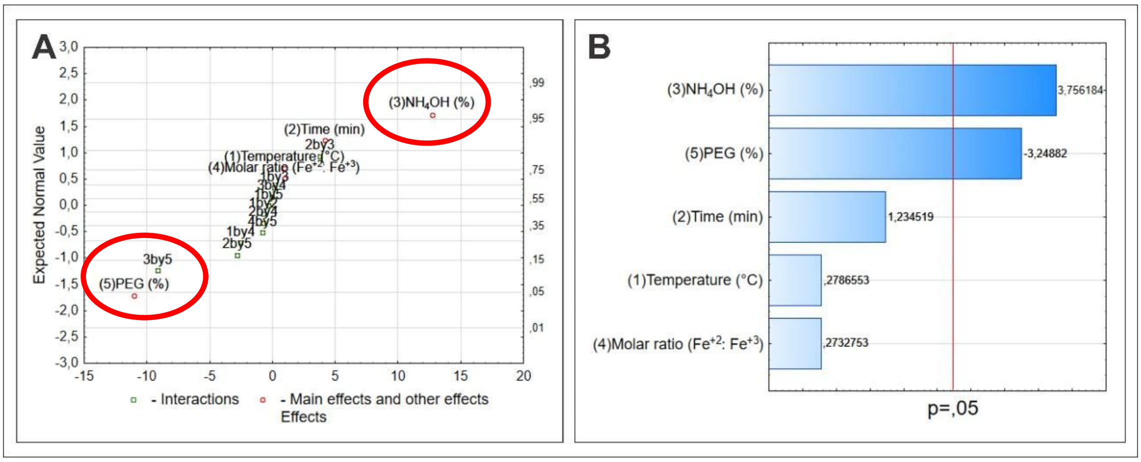

Figure S1. Normal effects graph of fractional factorial design (A). The Pareto bar chart plots the vertical lines that define the 0.05 significance level of the significant variables (B).

The results of the initial screening for the effective parameters in the synthesis reaction can also be observed in the analysis of variance shown in Table S2, which illustrates the statistical analysis of the variables NH_4OH and PEG concentration as the main effects. This information is necessary to construct the complete factorial design.

Table S2. ANOVA of the fractional factorial design of the magnetic nanoparticles.

	SS	df	MS	F	p
(1)Temperature (°C)	3.560	1	3.5599	0.07765	0.786187
(2)Time (min)	69.871	1	69.8709	1.52404	0.245232
(3)NH_4OH (%)	646.836	1	646.8360	14.10892	0.003746
(4)Molar ratio (Fe^{2+}: Fe^{3+})	3.424	1	3.4237	0.07468	0.790202
(5)PEG (%)	483.895	1	483.8947	10.55481	0.008738
Error	458.459	10	45.8459		
Total SS	1666.044	15			

*SQ – Sum of Squares; DF – Degrees of Freedom; MS – Mean Square; F – statistical test; P – p value; L – linear; Q – Quadratic. Significant variables are shown in bold.

Optimizing PEG and NH_4OH concentration

The optimal PEG and NH_4OH concentrations for production of the smallest nanomaterials were determined using the central composite design (CCD) design as shown in Table S3.

Table S3. Variables and levels used in the Central Composite Design (CCD) for optimizing the synthesis of the smallest nanomaterials

Factors	Levels		
	-1	0	+1
NH_4OH concentration (%)	4.0	16.0	28
PEG 4000 concentration (%)	0.5	5.5	10.5

* (−1) low values, (0) central values and (+1) high values.

The ANOVA test (Table S4) indicates a good fit of the model, due to the high F value and a very low probability value.

Table S4. ANOVA of the full factorial design of magnetic nanoparticles.

	SQ	df	MS	F	p
(1)PEG 4000 (%)(L)	192.1878	1	192.1878	37082.05	0.000027
PEG 4000 (%)(Q)	10.5185	1	10.5185	2029.51	0.000492
(2)NH_4OH (%)(L)	155.8403	1	155.8403	30068.92	0.000033
NH_4OH (%)(Q)	7.5299	1	7.5299	1452.88	0.000688
1L by 2L	92.9060	1	92.9060	17925.93	0.000056
1L by 2Q	0.0146	1	0.0146	2.81	0.235449
1Q by 2L	0.1962	1	0.1962	37.85	0.025418
1Q by 2Q	15.3379	1	15.3379	2959.41	0.000338
Error	0.0104	2	0.0052		
Total SS	504.9497	10			

*SQ – Sum of Squares; DF – Degrees of Freedom; MS – Mean Square; F – statistical test; P – p value; L – linear; Q – Quadratic. Significant variables are shown in bold.

The energy-dispersive X-ray spectroscopy (EDS) spectrum also presented additional peaks that were not discussed in the main text, as they were not relevant to the article's objectives. The focus of the analysis was to confirm the presence of iron and oxygen in the nanoparticles, as well as silicon from the TEOS used as a stabilizer. Unrelated peaks, such as carbon from the carbon tape used as a support and gold from the sample metallization process, were also detected. Although these secondary elements do not affect the analysis of the iron oxide nanoparticles' composition, their mention provides a more comprehensive understanding of the data. Overall, the EDS results confirm the presence of the key elements and the effectiveness of the stabilization strategy with TEOS.

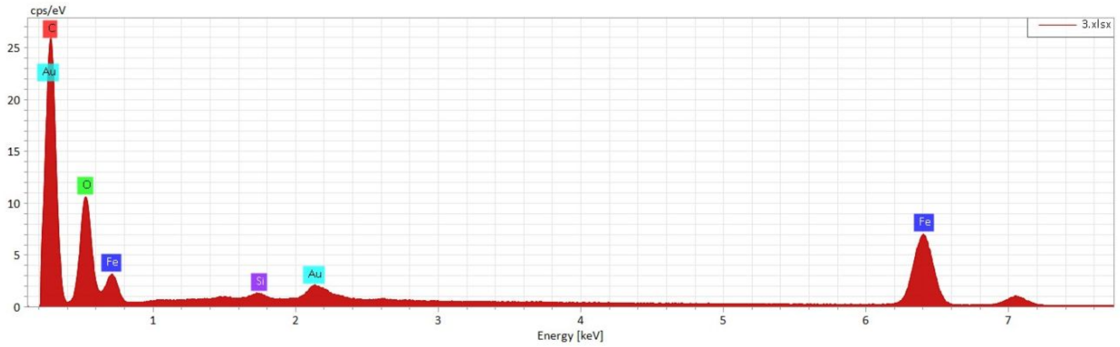

Figure S2. EDS analysis of TEOS-coated magnetic nanoparticles showing the main elements iron (Fe), oxygen (O), and silicon (Si). Additional peaks of carbon (C) and gold (Au) are attributed to supporting elements used in sample preparation.
